# The larva of *Drusus vinconi* Sipahiler, 1992 (Trichoptera, Limnephilidae, Drusinae)

**DOI:** 10.3897/zookeys.317.5749

**Published:** 2013-07-19

**Authors:** Johann Waringer, Wolfram Graf, Miklós Bálint, Mladen Kučinić, Steffen U. Pauls, Ana Previšić, Lujza Keresztes, Simon Vitecek

**Affiliations:** 1 Department of Limnology, Faculty of Life Sciences, University of Vienna, Austria; 2 Institute of Hydrobiology and Aquatic Ecology Management, University of Natural Resources and Applied Life Sciences, Vienna, Austria; 3 Biodiversity and Climate Research Centre (LOEWE BiK-F), Frankfurt a.M., Germany; 4 Department of Biology, Faculty of Science, University of Zagreb, Croatia; 5 Hungarian Department of Biology and Ecology, Babeş-Bolyai University, Cluj-Napoca, Romania

**Keywords:** *Drusus vinconi*, 5th instar larva, description, identification, distribution

## Abstract

This paper describes the previously unknown larva of *Drusus vinconi* Sipahiler, 1992. Information on the morphology of the 5th larval instar is given, and the most important diagnostic features are illustrated. In the context of existing identification keys the larva of *Drusus vinconi* keys together with *Drusus annulatus* (Stephens, 1837), *Drusus biguttatus* (Pictet, 1834), *Drusus ingridae* Sipahiler, 1993, *Hadimina torosensis* Sipahiler, 2002 and *Leptodrusus budtzi* (Ulmer, 1913). These species differ in the contours of the pronotum in lateral view, the presence/absence of the pronotal transverse groove, the shape of the median notch of the pronotum (in anterior view), pronotal sculpturing, presence/absence of the lateral carina of the head capsule, the number of proximo-dorsal setae on the mid-and hind femora, where the lateral fringe starts on the abdomen, and in geographic distribution. With respect to zoogeography, *Drusus vinconi* is a (micro-)endemic of the Western Pyrenees. The species prefers stony substratum in springs and springbrooks of the montane and subalpine region (Graf et al. 2008; Sipahiler 1992, 1993). As a grazer, the larvae of *Drusus vinconi* feed on biofilm and epilithic algae.

## Introduction

Extant Drusinae currently comprise 99 species. Thirty species are reported from the Alpine chain, another 34 species are known from the Balkan Peninsula (including many endemics). A total of 17 species have been described from south and southwestern Europe (Apennine, Iberia, Corsica, Pyrenees, southern France), and 18 species and 2 subspecies are known from Asia Minor and the Caucasus (Graf et al. 2008; Ivanov 2011; Malicky 2004, 2005; Oláh 2010, 2011; Sipahiler 2005;). However, the larvae of only 41 species (41%) have been described so far and included in keys (Botosaneanu 1959; Décamps and Pujol 1975; Despax 1927; Graf et al. 2011; Kučinić et al. 2008, 2010, 2011a, b; Moretti and Pirisinu 1981; Moretti 1983; Previšić et al. 2009; Sipahiler 2002; Szczesny 1978; Vieira-Lanero 2000; Vieira-Lanero et al. 2005; Waringer et al. 2008; Waringer and Graf 2011). To improve our knowledge of larval Drusinae taxonomy, we provide the description of the larva of *Drusus vinconi* Sipahiler, 1992 based on larval material collected in the Département Pyrénées-Atlantiques of the French Midi-Pyrénées region.

## Material and methods

Hand nets were used to collect larvae and adults of *Drusus vinconi* in and beside a small stream about 7 km SW of the ski area Arette La Pierre Saint Martin, Département Pyrénées-Atlantiques, Midi-Pyrénées, France (42°57'17.67"N, 0°49'26.91"W) on 23 July 2012 (leg. W. Graf). The material was preserved in 90% ethanol. A Nikon SMZ 1500 binocular microscope with DS-Fi1 camera and NIS-elements D 3.1 image stacking software for combining 8–50 frames in one focused image were used to study and photograph the larvae.

Species affiliation was enabled by the fact that putative *Drusus vinconi* larvae were collected close to their *locus typicus* where the only other Drusinae larvae present, *Drusus discolor* (Rambur, 1842), are clearly different from the species in question by their dense hair cover on head and pronotum. In addition, adults of both sexes of *Drusus vinconi* were collected at the same sites as the unknown larvae.

Deposition of voucher specimens: 2 5th instar larvae of *Drusus vinconi* are deposited in the collection of J. Waringer (Vienna, Austria) and 2 5th instar larvae and 1 male and 1 female in the collection of W. Graf (Vienna, Austria). Comparative material of other Drusinae included the following: *Drusus annulatus* (Stephens, 1837), 9 5th instar larvae; *Drusus biguttatus* (Pictet, 1834), 5 5th instar larvae; *Drusus ingridae* Sipahiler, 1993, 1 5th instar larva; *Leptodrusus budtzi* (Ulmer, 1913), 1 5th instar larva (all taxa: collection of J. Waringer, Vienna, Austria).

## Results

### Description of the 5th instar larva of *Drusus vinconi*

**Biometry.** Body length of 5th instar larvae ranging from 9.7 to 10.8 mm, head width from 1.76 to 1.90 mm (n = 2).

**Figures 1–6.**
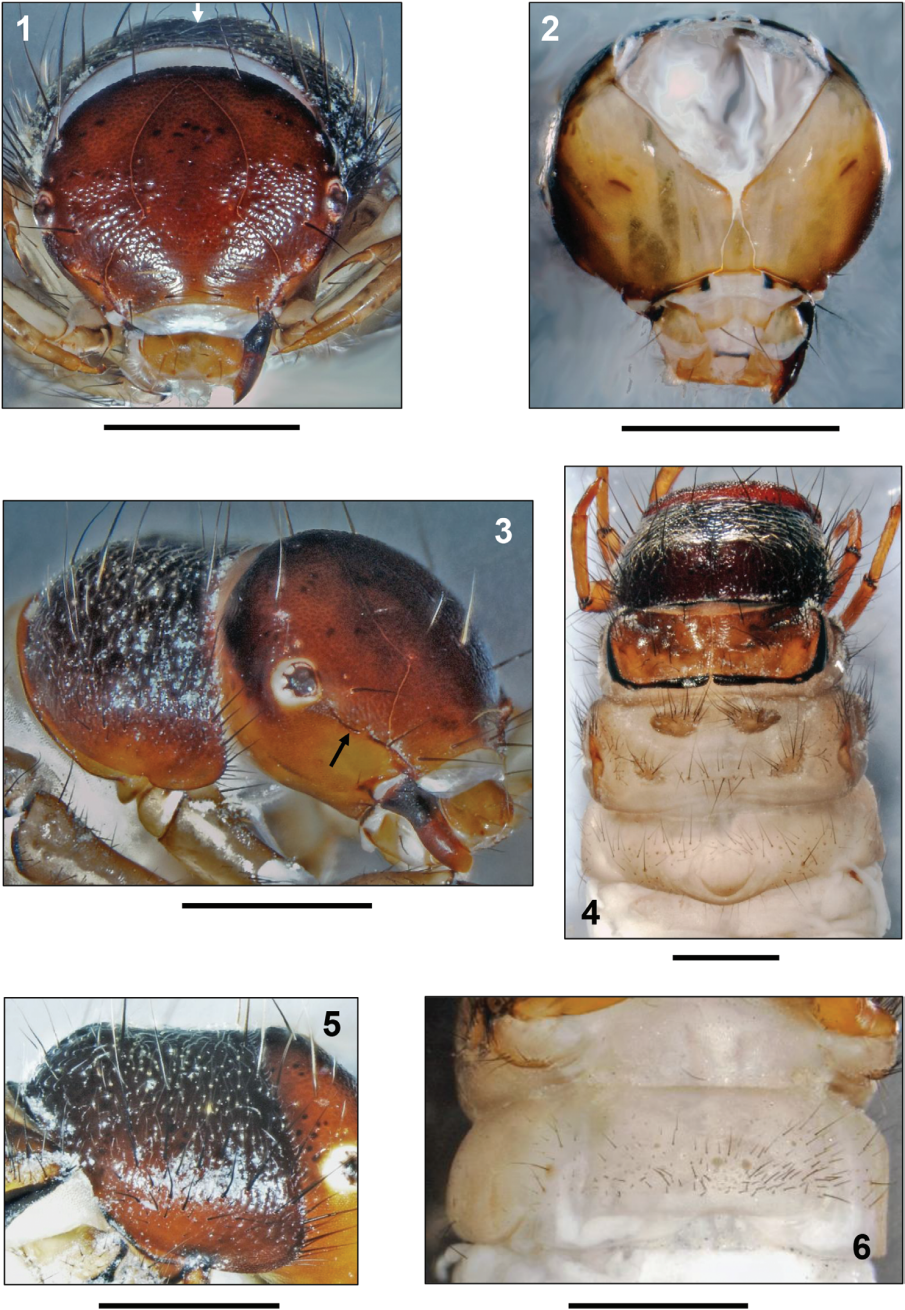
*Drusus vinconi* Sipahiler, 1992, 5th instar larva. **1** Head, dorsal view (arrow: median notch) **2** Head, ventral view **3** Head and prothorax, right lateral view (arrow: lateral carina) **4** Head, thorax and abdominal segment I, dorsal view **5** Pronotum, right lateral view **6** Abdominal sternum I, ventral view. Scale bars: 1 mm.

**Head.** Head capsule coarsely granulated, almost circular in shape, hypognathous (Figs 1, 3), dorsally chestnut to black brown, with blackish muscle attachment spots. Ventral parietalia sections, submentum, maxillolabial sclerites and premandibular areas yellowish (Figs 2, 3). Eyes surrounded by whitish ring (Fig. 3). In lateral view, head capsule bearing carina which extends from anterior eye margin to anterior corner of frontoclypeus (Fig. 3, black arrow). Complete set of 18 pairs of primary setae on head capsule (nomenclature *sensu* Wiggins 1998); no additional spines or spinule areas as known from other Drusinae larvae (e.g., *Ecclisopteryx* spp., *Drusus trifidus* McLachlan, 1868, most of the *Drusus bosnicus* group except *Drusus ramae* Marinković-Gospodnetić, 1971) present. Frontoclypeus bell-shaped, with narrow median constriction (Fig. 1). Antennae located dorsally on central section of lateral carinae (Fig. 3), each consisting of 1 short cylindrical base and 1 prominent lateral seta. On each parietal, 10 dorsal and 2 ventral primary setae present (Figs 1, 3). Each side of frontoclypeus bearing 6 primary setae, 3 of them along anterior border. Labrum yellowish brown, anterolateral margins with setal brush and primary setae 1–3; dorsally, setation consisting of primary setae 4–6 (Fig. 1). Yellow ventral apotome funnel-shaped with postgenal suture reaching approximately 29% of apotome length (Fig. 2). Black brown mandibles (sometimes brownish on distal half; Fig. 3) spoon-shaped, lacking terminal teeth along edges as well as ridges in central concavity (Figs 1, 3).

**Thorax.** Pronotum chestnut brown and very coarsely granulated, with adjacent series of granuli creating ribbed structures (Figs 3, 4). Posterior margin thickened and darkly striped; no pronotal transverse groove at end of anterior 3rd (Fig. 5). In lateral view, dorsal profile of pronotum low, with posterior 2/3rds being evenly rounded (Fig. 5). Along anterior pronotal border 2 setal rows present, including: i) dense fringe of short, curved, fine, yellow setae, ii) continuous row of widely-spaced long, straight, dark setae meeting at pronotal midline (Figs 1, 3, 4, 5). Each pronotal half bearing in total 35–45 dark setae of varying lengths. In addition, pronotal surface covered by high number of tiny, pale, curved, recumbent setae (Fig. 5); no spines as present in other Drusinae (e.g., *Drusus trifidus*). Prosternite inconspicuous, pentangular in shape, pale yellow, with light brown posterior border. Prosternal horn present (Fig. 3).

Mesonotum completely covered by 2 yellow brown to dark brown sclerites with anterolateral sections bearing darkest coloration. Median to dark brown muscle attachment spots present, lateral and posterior margins darkly sclerotized (Fig. 4). Counts for mesonotal setae (nomenclature *sensu* Wiggins 1998): anterior setal group *sa1*: 8–15, posterior group *sa2*: 25–30, lateral group *sa3*: 30–35 (Fig. 4). In addition, small number of tiny, pale, curved, recumbent setae present.

Metanotum partially covered by 3 pairs of yellowish grey sclerites (Fig. 4). Anterior metanotal sclerites (sclerites of setal area 1, *sa1*, *sensu* Wiggins 1998) very large, ovoid, tapering laterally. Medially, the 2 sclerites strongly divergent, widely spaced; their median separation nearly as high as their length along the longitudinal body axis (Fig. 4). Posteromedian sclerites (sclerites of setal area 2, *sa2*, *sensu* Wiggins 1998) small, triangular, with approximately 20 setae per sclerite, framing row of setae (Fig. 4). Lateral sclerites (sclerites of setal area 3, *sa3*, *sensu* Wiggins 1998) with approximately 25–30 setae concentrated in cranial section (Fig. 10). Groups of setae present between *sa2* and *sa3* (Fig. 4).

**Figures 7–13.**
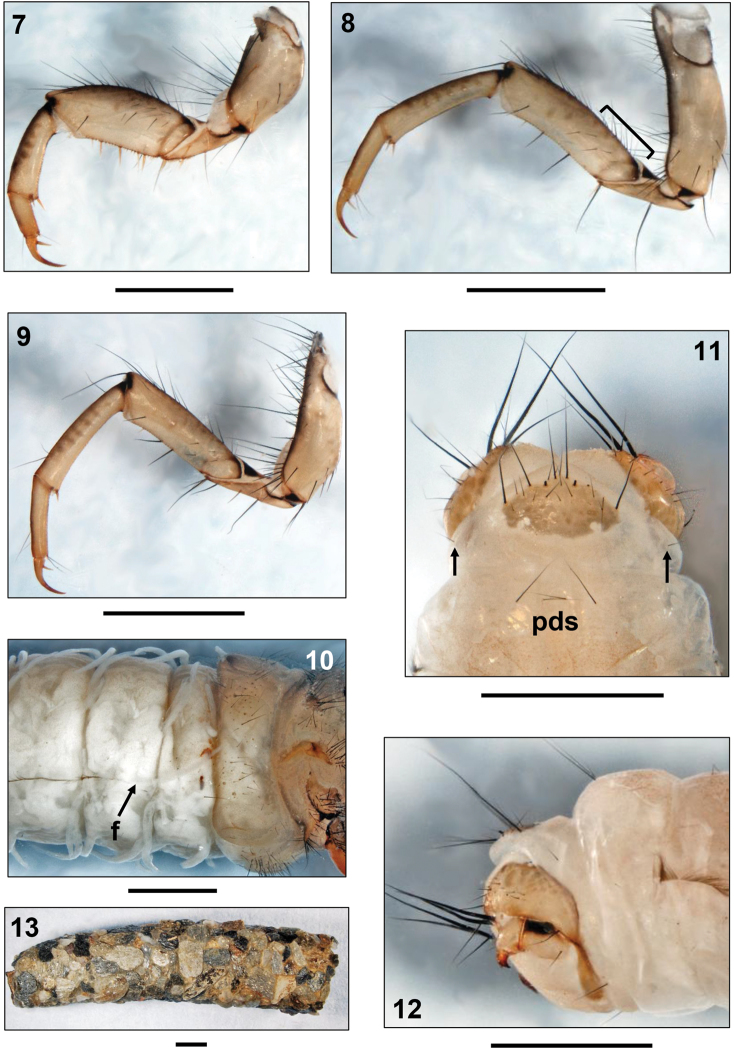
*Drusus vinconi* Sipahiler, 1992, 5th instar larva. **7** Right fore leg, anterior view **8** Right mid leg, anterior view (bracket: proximodorsal setae) **9** Right hind leg, anterior view **10** Metathorax and 1st 4 abdominal segments, right lateral view (f: start of lateral fringe at segment III) **11** Abdominal segments VIII-IX, dorsal view (arrows: posterolateral setae; pds: posterodorsal setae) **12** Apex of abdomen, right lateral view **13** Larval case, right lateral view. Scale bars: 1 mm.

Legs light brown with numerous setae on coxae, trochanters, and femora; tibiae and tarsi sparsely setose. Femora with several proximodorsal setae (e.g. Fig. 8, black bracket), and with setation on anterior and posterior faces; fore femora with 4, mid and hind femora with 3 yellow ventral-edge setae; no minute spines along ventral edges present. Foreleg coxa, femur and tibia wider than those of mid- and hind legs. Fore and mid trochanters with setae only on proximal sections; fore trochanters additionally with distal ventral trochanteral brush. Mid- and hind tibiae with dorsal setae only on distal 3rd (Figs 8, 9).

**Abdomen.** Abdominal segment I with 1 dorsal and 2 lateral fleshy protuberances (Figs 4, 10). Continuous transverse row of setae present anterior of dorsal protuberance (comprising fused setal areas *sa1*, *sa2*, *sa3*, *sensu* Wiggins 1998), stretching laterally from dorsal sections of lateral protuberances; posterior of dorsal protuberance, another row of setae present (Fig. 4). All these setae with small basal sclerites. Lateral protuberances without posterior sclerites (Fig. 10). Anterior of each lateral protuberance a continuous band of anterolateral setae connected to each dorsal and ventral *sa3* setal group (Fig. 10). Abdominal sternum I with fused setal areas *sa1*, *sa2* and *sa3*, creating continuous field of setae, therein occurs pair of central large basal sclerites with irregular borders and small number of randomly distributed basal sclerites of smaller diameter (Fig. 6). Abdominal dorsum VIII with 2 long and 2 short posterodorsal setae (pds) (Fig. 11 pds); only 1 posterolateral seta present on each half of abdominal dorsum IX (Fig. 11, arrows). Abdominal dorsum IX bearing beige pentangular sclerite with 8 long and several short setae (Fig. 11). Beige anal prolegs are of limnephilid type with medium brown anal claws, each with 1 small accessory hook (Fig. 12).

All gills as single filaments (Fig. 10). Dorsal gills present at most from abdominal segments II-VII (presegmental positions). Ventral gills present from segment II (presegmental) to segment VII (postsegmental). In lateral row, gills present on segments II-III only (ventrolateral position). Lateral fringe extends from anterior border of segment III (Fig. 10f) to middle of segment VIII.

**Case.** Larval case 8.5–12.1 mm long (n= 2), curved, conical (width at anterior opening 2.9–3.2 mm, at posterior opening 1.9–2.2 mm), consisting of mineral particles (sand grains of mixed size; Fig. 13).

### Morphological separation of 5th instar larvae of *Drusus vinconi* from other European Trichoptera

Within the framework of the larval key by Waringer and Graf (2011), *Drusinae* larvae are separated from other Trichoptera species by the following features:

- sclerites present on pro-, meso- and metanota; mesontum completely covered by 2 sclerites in close contact separated by a straight suture; metanotum incompletely sclerotized by 6 sclerites (Fig. 4);
- prosternal horn present (Fig. 3);
- fleshy protuberances at abdominal segment I present dorsally and ventrally (Figs 4, 10);
- gills consisting of single filaments only (Fig. 10);
- transverse groove lacking at the anterior 3rd of the pronotum (Fig. 5) except in *Leptodrusus budtzi* (Fig. 16).

Within the subfamily Drusinae, *Drusus vinconi* is characterised by the following set of morphological details:

- mandibles spoon-shaped (Figs 1, 3);
- head capsule without additional spines or spinules (Fig. 1);
- anterior-row setae present near dorsal pronotal midline (Figs 1, 3);
- dorsal gills present (Fig. 10);
- dorsal edge setae restricted to distal 3rd of mid and hind tibiae (Figs 8, 9);
- basal sclerites of setae on abdominal sternum I separated (Fig. 6);
- pronotum evenly rounded (Fig. 5).

**Figures 14–19.**
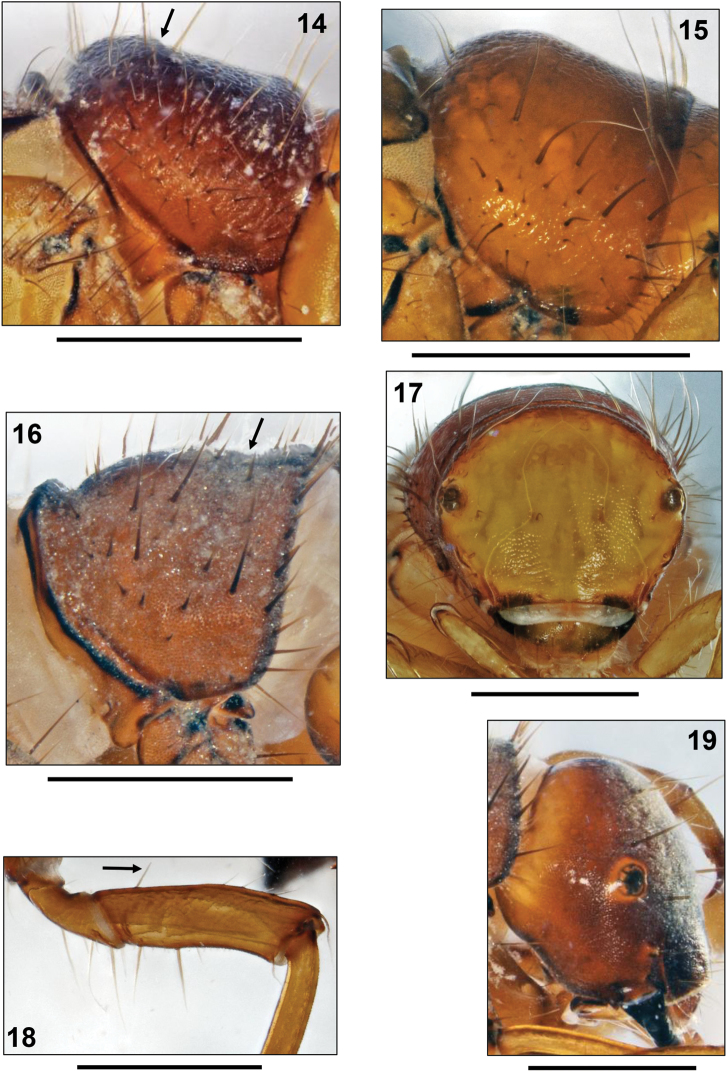
**14–16** Pronota of 5th instar larvae, right lateral views. **14** *Drusus annulatus* (Stephens, 1837) (arrow: dorsal profile angled) **15** *Drusus biguttatus* (Pictet, 1834) **16** *Leptodrusus budtzi* (Ulmer, 1913) (arrow: transverse groove) **17** *Drusus biguttatus*, head of 5th instar larva, frontal view. **18–19** *Leptodrusus budtzi*, 5th instar larva **18** Left midleg, posterior view (arrow: proximodorsal seta) **19** Head, right lateral view. Scale bars: 1 mm.

At this position in the key, *Drusus vinconi* appears together with *Drusus annulatus*, *Drusus biguttatus* (Pictet, 1834), *Drusus ingridae*, *Hadimina torosensis* Sipahiler, 2002 and *Leptodrusus budtzi*. These species are easily distinguished by differences in dorsal profile, presence/absence of the lateral carina on the head capsule, number of proximo-dorsal setae on mid-and hind femora, origin of abdominal lateral fringe, and geographic distribution (Table 1).

**Table 1.** Synopsis of characters separating the currently known Drusinae larvae (5th instars) which share the following morphomatrix: spoon-shaped mandibles; lack of additional head spines or spinules; anterior-row setae present near dorsal pronotal midline; dorsal gills present; dorsal edge setae restricted to distal third of mid and hind tibiae; basal sclerites of setae at first abdominal sternum separated; pronotum evenly rounded. Data for *Hadimina torosensis* were taken from Sipahiler (2002).

**Species /**<br/> **character**	**Dorsal outline of pronotum (lateral view)**	**Pronotal transverse groove at end of anterior 3^rd^ present?**	**Pronotum with median notch (anterior view)?**	**Pronotal sculpturing / cover of procumbent pale setae**	**Head capsule with lateral carina?**	**More than one proximo-dorsal seta on mid-and hind femora?**	**Start of lateral fringe**	**Distribution**
*Drusus annulatus*	angled (Fig. 14)	no	no	coarsely<br/> granulated /<br/> sparse	yes	yes	first third III	widespread
*Drusus biguttatus*	evenly rounded,<br/> high profile<br/> (Fig. 15)	no	no<br/> (Fig. 17)	coarsely<br/> granulated /<br/> sparse	yes	yes	last third II	widespread
*Drusus ingridae*	evenly rounded,<br/> low profile	no	no	coarsely<br/> granulated /<br/> sparse	yes	yes	first third III	Pyrenees,<br/> Massif Central
*Drusus vinconi*	evenly rounded,<br/> low profile<br/> (Fig. 5)	no<br/> (Fig. 5)	yes<br/> (Fig. 1)	coarsely<br/> granulated /<br/> dense	yes<br/> (Fig. 3)	yes<br/> (Figs 8, 9)	first third III<br/> (Fig. 10)	Pyrenees
*Hadimina torosensis*	evenly rounded,<br/> high profile	no	?	? / ?	yes	yes	first third II	Asia Minor
*Leptodrusus budtzi*	evenly rounded,<br/> low profile<br/> (Fig. 16)	yes (Fig. 16)	no	finely<br/> granulated / sparse (Fig. 16)	no<br/> (Fig. 19)	no<br/> (Fig. 18)	last third II	Corsica,<br/> Sardinia, Mallorca

## Discussion

*Drusus vinconi* is a (micro-)endemic of the Western Pyrenees. Its *locus typicus* is situated at the ruisseau de Chousse, a tributary of the Vert d’Arette, near the Serre de Benou, at 1300 m a.s.l. At this site *Drusus discolor* was the only other Drusinae species. Larvae of *Drusus discolor* are clearly different from *Drusus vinconi* larvae by their dense hair cover on the head and pronotum.

Adults of *Drusus vinconi* are morphologically close to *Drusus monticola* McLachlan, 1876. Differences exist in the structure of the male intermediate appendages which are triangular, and in the preanal appendages which are long and ovoid in *Drusus vinconi*. The female is characterised by a very short median scale (Sipahiler 1992).

The species was abundant in a small, stony stream near the ski area Arette La Pierre St Martin in the Département Pyrénées-Atlantiques of the Midi-Pyrénées region, France. *Drusus vinconi* is a rheophilic species inhabiting springs and springbrooks where it can be observed on the surface of boulders and large stones (Graf et al. 2008). According to its mouthpart anatomy, *Drusus vinconi* is a grazer, feeding exclusively on epilithic algae and biofilm. Records exist from montane and subalpine sites situated well above 800 m a.s.l. (Sipahiler 1992, 1993). Adults fly in June and July.
